# Diverse associations observed between pregnancy complications and RBC or plasma folates determined by an in-house developed LC-MS/MS method

**DOI:** 10.1080/07853890.2023.2265381

**Published:** 2023-10-12

**Authors:** Zhengwen Xu, Youran Li, Ying Liu, Shengmin Liu, Lin Zhang, Jing Wang, Shaofei Su, Lin Liu, Lanlan Meng, Hongyuan Zhu, Jingru Sun, Lijun Shao, Lin Li, Yanhong Zhai, Guanghui Li, Zheng Cao

**Affiliations:** a Department of Laboratory Medicine, Beijing Obstetrics and Gynecology Hospital, Capital Medical University, Beijing Maternal and Child Health Care Hospital, Beijing, PR China; b Center of Clinical Mass Spectrometry, Beijing Obstetrics and Gynecology Hospital, Capital Medical University, Beijing Maternal and Child Health Care Hospital, Beijing, PR China; c Health Biotech Co. Ltd, Beijing, PR China; d Department of Internal Medicine, Beijing Obstetrics and Gynecology Hospital, Capital Medical University, Beijing Maternal and Child Health Care Hospital, Beijing, PR China; e Central Laboratory, Beijing Obstetrics and Gynecology Hospital, Capital Medical University, Beijing Maternal and Child Health Care Hospital, Beijing, PR China; f Department of Obstetrics, Beijing Obstetrics and Gynecology Hospital, Division of Endocrinology and Metabolism, Capital Medical University, Beijing Maternal and Child Health Care Hospital, Beijing, PR China

**Keywords:** Folate, plasma, RBC, LC-MS/MS, gestational diabetes mellitus, gestational hypertension, preeclampsia

## Abstract

**Background:**

As folates are essential for embryonic development and growth, it is necessary to accurately determine the levels of folates in plasma and red blood cells (RBCs) for clinical intervention. The aims of this study were to develop and validate a liquid chromatography-tandem mass spectrometry (LC-MS/MS) method for quantitation of folates in plasma and RBCs and to examine the association between plasma and RBC folate concentrations and gestational diabetes mellitus (GDM), gestational hypertension (GH) and preeclampsia (PE).

**Methods:**

With the in-house developed LC-MS/MS, a retrospective cross-sectional study was conducted. The healthy pregnant women of first- (*n* = 147), second- (*n* = 84) and third-trimester (*n* = 141) or the women diagnosed with GDM (*n* = 84), GH (*n* = 58) or PE (*n* = 23), that were aged between 22 and 46 years old and registered at our institute, were subjected for measurement of folic acid (FA) and 5-methyltetrahydrofolate (5-MTHF), followed by appropriate statistical association analysis.

**Results:**

The assay for simultaneous quantitation of FA and 5-MTHF in plasma and RBCs was linear, stable, with imprecision less than 15% and recoveries within ±10%. The lower limits of quantification for FA and 5-MTHF measurement in whole blood were 0.57 and 1.09 nmol/L, and in plasma were 0.5 and 1 nmol/L, respectively. In the association analysis, the patients with lower RBC folate level (<906 nmol/L) presented higher risks of PE development (OR 4.861 [95% CI 1.411–16.505]) by logistic regression and restricted cubic spline (RCS) regression in a nonlinear fashion. In addition, higher level of plasma folates in pregnancy was significantly associated with GH risk but may be protective for the development of GDM.

**Conclusions:**

The in-house developed LC-MS/MS method for folates and metabolites in plasma or RBC showed satisfactory analytical performance for clinical application. Further, the levels of folates and metabolites were diversely associated with GDM, GH and PE development.

## Introduction

Folate is an essential water-soluble B vitamin (B9) [1] that is not synthesized in mammalian cells and can only be obtained by diet or from supplementation [2]. As a crucial nutrient for normal reproduction and pregnancy, women in many countries are recommended to start folic acid (FA) supplementation from one month before conception and throughout the first trimester of pregnancy due to the increased physiological need for this vitamin to support rapid fetal growth and placental development [3–5]. Studies have shown pregnant women are more likely to develop FA deficiency that ultimately leads to fetal neural tube defects (NTDs) [6], megaloblastic anemia [7], and adverse pregnancy outcomes, such as premature delivery, fetal growth restriction and extremely low birth weight [8].

Thus, the assessment of folate status in pregnant population is highly clinical-concerned [9,10]. Both red blood cell (RBC) and serum or plasma folates are related to folate intake, but they belong to different biological processes and cannot convert to each other [11,12]. It is generally accepted that serum or plasma folate is a short-term indicator for recent dietary intake, whereas RBC folate more accurately reflects long-term liver storage [13,14]. As folate is only taken up by RBCs during erythropoiesis and the life span of red cell folate closely matches that of RBCs (about 120 d) [15], therefore, it would take 4 half-lives for blood concentrations to reach 94% of a new steady state if the intake changes [16]. The concentration of folates in RBCs changes relatively slowly with the alteration of intake and stable levels may last for 35 weeks. From a public health perspective, RBC folate has indispensable clinical significance and can identify women at risk of pregnancy complications who may benefit from targeted dietary advice and additional folate supplementation [17].

In clinical laboratories, the main-stream methods for folates measurement are chemiluminescence or electrochemiluminescence-based competitive-binding receptor assay involving the usage of folate binding protein (FBP) [18]. As FBPs have different affinities between polyglutamate and monoglutamate folate species, this assay is easily affected by the diversity of folate forms that occur naturally [19,20]. The application of liquid chromatographic tandem mass spectrometric (LC–MS/MS) brings up advantages of discriminating between different metabolically active folate metabolites with exceptional speed, sensitivity and quantitation ability [21]. In this study, we established a simple and reliable LC–MS/MS method for determination of folates in plasma and RBCs. The associations between plasma or RBC folate levels and common pregnancy complications, such as gestational diabetes mellitus (GDM), gestational hypertension (GH) and preeclampsia (PE), were explored statistically.

## Materials and methods

### Subjects

In this single‑center, retrospective cross‑sectional study, the complete blood count (CBC) leftover specimens were obtained from the pregnant women registered at Beijing Obstetrics and Gynecology Hospital. For the folates evaluation in healthy pregnant women, from February 2022 to May 2022, totally 800 CBC leftover specimens were initially collected and stored at −80 °C until LC-MS/MS analysis [first trimester (*n* = 300), second trimester (*n* = 200) and third trimester (*n* = 300)]. The healthy subjects selection criteria were as follows [22,23]: (1) with essentially normal antenatal laboratory workup throughout pregnancy; (2) with no history of mental illness and other serious diseases; (3) with uncomplicated previous pregnancies and deliveries; (4) without any pregnancy complications or adverse pregnancy outcomes, such as GDM, GH, PE, oligohydramnios and polyhydramnios, or their infants with premature birth, low birth weight, and macrosomia. After applying the selection criteria, there were total 372 healthy pregnant women (aged between 22 and 46 years) were enrolled in our folate evaluation, including 147 in the first trimester (6 − 11 gestational weeks), 84 in the second trimester (24 − 28 gestational weeks) and 141 in the third trimester (28 − 40 gestational weeks). Furthermore, we retrospectively collected the CBC leftover specimens from the patients diagnosed with GDM, GH and PE from February 2022 to November 2022. And a total of 165 pregnant women with pregnancy complications were included in this study, including GDM (*n* = 84), GH (*n* = 58) and PE (*n* = 23). The clinical diagnosis criteria for the above complications are listed in Supplementary Table 1.

### Chemicals and reagents

The two folate monoglutamates investigated in this study were FA and methyl-tetrahydro folic acid (5-MTHF). The chemical standards and isotype-labeled internal standards (IS) of FA (IS: ^13^C_5_-FA) and 5-MTHF (IS: ^13^C_5_-5-MTHF) were acquired from Merck KGaA (Darmstadt, German) and Toronto Research Chemical (Toronto, Canada), respectively. Methanol (Optima^®^ LC/MS grade) formic acid (LC/MS grade, 98%), and acetonitrile (Optima^®^ LC/MS grade) were purchased from Fisher Scientific (Fair Lawn, USA). Acetate (AR, 99.5%) was purchased from Aladdin (Shanghai, China). Ammonia solution (contains ≥25 − 30%) and ascorbic acid (99%) were acquired from Merck KGaA (Darmstadt, Germany).

### Preparation of calibrators, quality control samples and internal standards

The stock solutions (0.1 mg/mL) were prepared by dissolving standards and adding ascorbic acid powder in degassed 20 mM phosphate buffer (pH = 7.2). The mixed working solution series were obtained by spiking an appropriate amount of each stock solution into 1% ascorbic acid. The six-point calibrators prepared by spiking mixed working solutions into 0.1% ascorbic acid and the resulting concentrations are listed in Supplementary Table 2. The working internal standard solution was prepared with 1% ascorbic acid as a mixture; the working concentrations used was 100 ng/mL for ^13^C_5_-FA or ^13^C_5_-5-MTHF.

### Sample preparation

The whole blood samples were collected from the participants who underwent CBC analysis in the Obstetrics Department at our institute from February 2022 to November 2022. Plasma samples were obtained by centrifugating the whole blood samples at 3000 rpm for 10 min. The solid phase extraction (SPE) method was applied to extract folate derivatives from whole blood and plasma. Briefly, 180 μL of protective agent (1% w/v ascorbic acid) was added to 20 μL whole blood or plasma samples, followed by addition of 50 μL of internal standard solution (100 ng/mL). Then, the mixtures were incubated at 37 °C for 4 h for folate deconjugation. The remaining samples were further purified using the complementary retention cartridges (SOLA HRP) in a 96-well format with a 10 mg/2 mL reversed polymer (Thermo Fisher Scientific, Am parir 20, Langerwehe, Germany). Before usage, the SPE cartridge was successively equilibrated with 1 ml of methanol, acetonitrile, and the solvent 1 (1% w/v ammonium mixed with 1% w/v ascorbic acid). After the addition of 800 μl solvent 1, the sample mixture was loaded onto the equilibrated SPE cartridges and washed twice with 1 mL of solvent 2 (0.05% w/v ammonium mixed containing 0.1% w/v ascorbic acid). Finally, the extracted folate derivatives were eluted with 0.2 mL of elution solution (20% v/v methanol, 10% v/v acetonitrile and 1% v/v acetate containing 0.5% w/v ascorbic acid) and were ready for the subsequent LC-MS/MS injection and analysis.

### Instrumentation and conditions

The quantitative analysis of folates was performed on a HPLC-MS/MS system consisting of a high-performance liquid chromatography (HPLC) coupled with a 4500 MD (AB Sciex, Framingham, MA) triple quadrupole mass spectrometer equipped with an electrospray ionization probe. Chromatographic separation was completed using an ACQUITY UPLC^®^HSS T3 column (1.8 µm 2.1 × 50 mm) from Waters (Milford, CT). The gradient elution with a flow rate of 0.35 mL/min was carried out using 0.1% formic acid in water (v/v) as mobile phase A and acetonitrile as mobile phase B. The elution program was set as follows (for a total run-time of 4.0 min with 10 μL single injection volume): 20% B for 0 − 2.0 min, 20 − 60% B for 2.0 − 2.5 min, 60% B for 2.5 − 3.0 min, 60 − 20% B for 3.0 − 4.0 min. The column oven and auto-sampler temperatures were set to 40 °C and 15 °C, respectively.

Positive electrospray ionization was used for the detection of folate metabolites. Two specific mass transitions (*m/z*) were chosen for each compound using the multiple reaction monitoring mode (MRM) function (one transition for quantitation, the other transition for qualification). The optimized compound-dependent mass parameters and the MRM transitions are listed in Supplementary Table 3.

### Assay validation

The developed method was validated according to the recommendations published in the Clinical and Laboratory Standards Institute (CLSI) C62-A. The method validation included examining the following analytical aspects of this assay: linearity, lower limit of quantification (LLOQ), imprecision (intra-assay/inter-assay), accuracy, matrix effect, carry over and stability.

The actual LLOQ was verified as the concentration with a % bias and relative standard deviation (RSD) below 20%. Duplicate calibration curves of 6 calibrators for plasma or whole blood were constructed on 3 non-consecutive days to evaluate linearity. A weighted least-squares regression model was used to check the linearity of the method. Specifically, the linearity was evaluated using the 6-point calibrators by measuring the ratio of analyte peak area to IS area against the nominal concentrations. A correlation coefficient (*R*) of 0.99 or higher was considered acceptable.

Quality control (QC) samples prepared at three different concentrations were used to measure the intra- and inter-assay precision. The intra-assay precision was determined by running each QC sample in six replicates on the same day, and the inter-assay precision was determined by running QC samples on three successive days. The inter- and intra-day precision values were expressed as the coefficient of variation (CV).

The carry over was assessed by alternating high-level QC samples and blank samples five times. Any peak that matches the target analytes and is found in the blank samples should be below the LOD.

The accuracy was evaluated by the recovery studies, in which three levels of calibrators with different concentrations were added to the pool of pregnant specimens. The recovery rate was calculated at high-, medium- and low-level QCs by comparing the ratio of the measured concentration with the deduction of background value to the expected concentration.

The matrix effect was estimated by comparing the peak response of the post-extraction spiked plasma or whole blood with the neat solvent standards at the three QC concentrations.

The stability was assessed by reinjection of samples stored for 20 h in the autosampler while concentrations were calculated based on fresh calibrators. Long-term stability studies were performed using spiked plasma or whole blood samples at low and high QC concentrations (*n* = 6) at −20 °C.

### Statistical analysis and calculation

The Kruskal − Wallis test was used to examine statistical differences in the levels of folate among women in three trimesters of normal pregnancy. The differences of folate levels between disease and healthy groups were evaluated using the Mann–Whitney U test for non-normally distributed continuous variables. All statistical analyses were performed with the SPSS version 26.0 software (IBM Corporation, New York, NY). The significance level was *p* < 0.05. The RBC folate concentrations higher than 400 ng/mL (906 nmol/L) have been recommended by WHO as the optimal cutoff level in women of reproductive age [24]. Additionally, the RBC folate concentrations greater than 600 ng/mL (1360 nmol/L) was considered as high RBC folate [25]. Accordingly, the RBC folate concentrations were categorized as < 906, 906–1360 and ≥1360 nmol/L in this study. Plasma folate was categorized into tertiles as there is no specified cutoff for pregnancy by literature review. Multivariable logistic regression analyses were conducted to investigate the associations between RBC or plasma folate levels and pregnancy complications including GDM, GH and PE, with adjustment for age. Moreover, restricted cubic spline (RCS) regression model with assumed three knots was used to outline the potential nonlinear relations between continuous RBC folates and PE risk. Stata version 16.0 (StataCorp LLC, College Station, TX) was used for logistic regression analysis. And two-tailed *p* value of 0.05 was defined as statistical significance.

### Ethical approval

The study was approved by the Ethics Committee of the Beijing Obstetrics and Gynecology Hospital (2022-KY-021-01). The need for informed consent from included individuals was waived by the Ethics Committee as the leftover blood samples from routine antenatal check-up were used in this study. The laboratory and medical data were de-identified before use.

## Results

### Assay validation for LC-MS/MS method

The FA and 5-MTHF extracted from whole blood or plasma were detected with suitable separation and symmetrical peaks, with representative chromatograms presented in Supplementary Figure 1. To examine the analytical performance of the proposed method, its linearity, LLOQ and imprecision (intra-assay/inter-assay), accuracy, matrix effect, carry over and stability were critically evaluated.

Over the calibration range selected, the method was found linear with the coefficient values of *R* > 0.995 for the two analytes both in whole blood and plasma. The slopes and intercepts of the linear regression equations for each analyte are listed in Table 1. The linear ranges of the method were as follows: 0.57 − 56.75 and 1.09 − 108.75 nmol/L for FA and 5-MTHF in whole blood, 0.5 − 50 and 1 − 100 nmol/L for FA and 5-MTHF in plasma. The LLOQ for FA and 5-MTHF measurement in whole blood were 0.57 and 1.09 nmol/L, and in plasma were 0.5 and 1 nmol/L, respectively (Table 1). The FA and 5-MTHF extracted from the whole blood and plasma samples were stable for 20 h at 15 °C and for 30 d at −20 °C (Table 1).

**Table 1.** The linearity, LLOQ and stability in assay validation.

		Linear regression		LLOQ (nmol /L)	Stability, %	
Analytes	*R*	Slope (±SD)	Intercept (±SD)		15 °C	−20 °C
FA^a^	0.996	0.0067 ± 0.0012	0.0041 ± 0.0004	0.57	105.91	98.89
5-MTHF^a^	0.995	0.0420 ± 0.0036	0.0052 ± 0.0024	1.09	101.72	101
FA^b^	0.996	0.0055 ± 0.0009	0.0051 ± 0.0008	0.50	101.41	98.13
5-MTHF^b^	0.996	0.0203 ± 0.0021	0.0037 ± 0.0003	1.00	101.06	96.97

SD: standard deviation

Stability: recovery rates for plasma samples stored at 15 °C for 20 h and at −20 °C for one month. ^a^In whole blood; ^b^in plasma.

The intra-assay and inter-assay precision were assessed at the three QC levels. The imprecision was ranged from 4.3 to 6.5% (FA) and 4.9 to 8.4% (5-MTHF) in plasma, and from 1.6 to 14.9% (FA) and 4.8 to 12.1% (5-MTHF) in whole blood (Table 2). The accuracy measured by % bias was between 92.1 and 107.9% at QC-L, QC-M and QC-H levels, better than ±20% acceptance criteria (Table 2). The carry over for the two analytes in whole blood and plasma were acceptable (< LOD in the blank sample) (data not shown).

**Table 2.** The recoveries and imprecisions in assay validation.

	FA^a^	5-MTHF^a^	FA^b^	5-MTHF^b^
Recovery, %				
QC-L	96.9	107.9	95.7	100.1
QC-M	92.1	93.3	98.9	99.6
QC-H	95.8	97.2	97.6	98.4
Intra-assay CV, %				
QC-L	14.9	8.5	6.5	4.3
QC-M	3.5	1.6	4.4	5.6
QC-H	4.9	3.0	5.0	4.3
Inter-assay CV, %				
QC-L	12.1	11.5	7.0	8.4
QC-M	8.4	6.6	6.5	7.5
QC-H	6.4	4.8	4.9	5.8

QC-L: low-level quality control; QC-M: medium-level quality control; QC-H: high-level quality control.

^a^In whole blood; ^b^in plasma.

In the matrix effect evaluation, the recovery rates for FA and 5-MTHF were 91.2% and 103.9% in whole blood, and were 95.1% and 102.0% in plasma, respectively.

### Associations between folate metabolites and pregnancy complications

**Figure 1.**
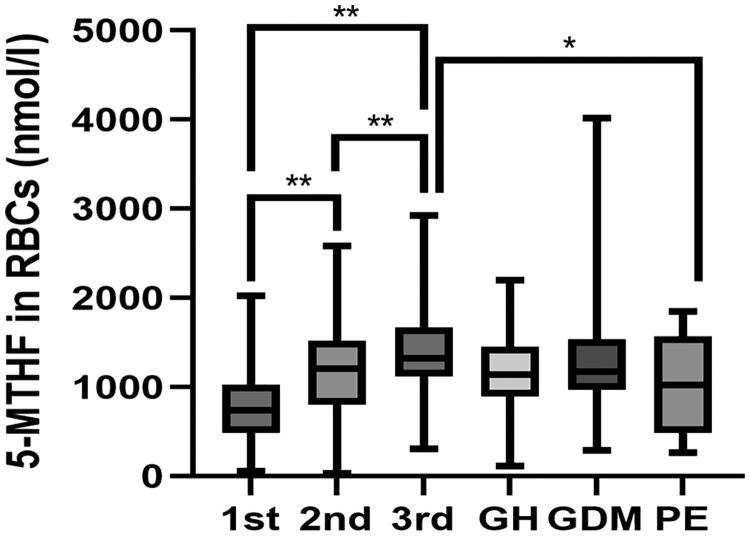
Box plots representing the 5-MTHF levels in RBCs between normal pregnancy and pregnant women with pregnancy complications. *Indicates *p* < 0.05, **indicates *p* < 0.01. GH: gestational hypertension; GDM: gestational diabetes mellitus; PE: preeclampsia; 5-MTHF: 5-methyltetrahydrofolate; RBCs: red blood cells.

The levels of FA and 5-MTHF in RBCs showed continuous elevation throughout pregnancy, displaying significant difference among different trimesters in normal pregnant women (Kruskal − Wallis test, *p* < 0.01) (Figure 1 and Table 3). By contrast, no significant difference of FA and 5-MTHF in plasma was found as pregnancy advanced chronically (*p* > 0.05). The levels of FA in RBCs were higher in the GDM group than the non-GDM controls that were tested between 24 and 28 GWs of pregnancy. In addition, the levels of 5-MTHF in RBCs were significantly decreased in preeclamptic mothers as compared to the GW-matched healthy controls (*p* < 0.05, Figure 1 and Table 3). As for the folate metabolites in plasma, the level of FA in plasma was lower in maternal blood of pregnant women with PE when compared to that of the normotensive pregnant women in the third trimester (*p* < 0.05). A lower level of 5-MTHF in plasma was observed in the women with GDM than that in normal pregnancy. However, no significant association of folates in RBCs or plasma was found between the GH group and control group.

**Table 3.** Distribution of 5-MTHF, FA and total folate levels in plasma and RBCs and difference between blood sampling periods.

	1st	2nd	3rd
FA^a^ (nmol/L)	0.80 (0.50 − 10.49)	0.78 (0.12 − 2.64)	0.83 (0.5 − 19.58)
5-MTHF^a^ (nmol/L)	49.30 (14.89 − 79.98)	49.90 (9.95 − 78.24)	53 (11.66 − 83.52)
plasma folates (nmol/L)	48.95 (12.99 − 79.61)	50.52 (9.86 − 79.31)	54.02 (12.16 − 92.67)
FA^b^ (nmol/L)	4.78 (0.16 − 25.89)	14.62 (3.32 − 53.81)	21.05 (1.30 − 257.93)
5-MTHF^b^ (nmol/L)	743.06 (117.07 − 1677.48)	1215.97 (103.35 − 2294.19)	1321.17 (435.58 − 2388.88)
RBC folates (nmol/L)	764.74 (122.88 − 1678.31)	1229.52 (145.83 − 2314.46)	1383.33 (471.48 − 2457.68)
	GH	GDM	PE
FA^a^ (nmol/L)	0.77 (0.17 − 5.84)	0.74 (0.14 − 3.22)	0.56 (0.10 − 2.64)
5-MTHF^a^ (nmol/L)	50 (17.63 − 109.88)	42.75 (14.61 − 79.96)	43.90 (8.14 − 89.64)
plasma folates (nmol/L)	50.76 (17.79 − 111.61)	43.56 (15.66 − 81.36)	44.08 (8.27 − 90.38)
FA^b^ (nmol/L)	19.42 (0.44 − 63.27)	18.68 (1.49 − 75.18)	22.50 (1.67 − 68.41)
5-MTHF^b^ (nmol/L)	1128.47 (148.80 − 2181.21)	1172.20 (319.04 − 2358.46)	1026.28 (266.15 − 1845.50)
RBC folates (nmol/L)	1159.97 (160.69 − 2190.14)	1200.86 (340.89 − 2371.11)	1087.72 (273.69 − 1867.12)

^a^In plasma; ^b^in RBCs.

**Figure 2.**
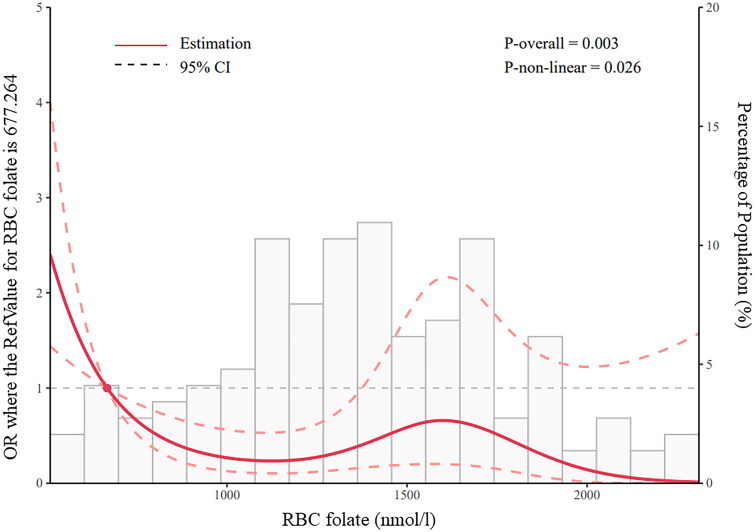
Restricted cubic spline (RCS) regression analysis of RBC folate with PE risk. RBC folate 677.264 nmol/L was selected as the reference value, when or = 1.0. The solid line indicates estimated ORs, and the areas between the dashed lines represent 95% CI. P-overall = 0.003, P-non-linear = 0.026.

In the association study, a significant and negative correlation was identified between the levels of RBC folates and the development of PE. Compared with the pregnant women with RBC folate levels between 906 and 1360 nmol/L, those with RBC folate lower than 906 nmol/L were associated with almost 5-fold higher odds of PE (OR 4.861 [95% CI 1.411–16.505]) (*p* = 0.011, Table 4). A similar trend was also found in the plasma folates of pregnant women with PE. The risk of PE became significantly higher in Q1 tertile compared with that in Q2 tertile (OR 3.44 [95% CI 1.254–9.434]; *p* = 0.016). Consistently, the RCS regression model revealed that lower levels of RBC folate were associated with an increased risk of PE in a nonlinear fashion (Figure 2), implying the importance of folate supplementation in PE prevention. No statistically significant association was identified between RBC folates and GH or GDM. Interestingly, the risk of GH was higher in Q3 tertile compared with that in Q2 tertile (OR 2.274 [95% CI 1.11–4.657]; *p* = 0.025), indicating that increased level of plasma folates (or recent dietary intake) in pregnancy was positively associated with GH development (Table 4). On the contrary, the women with high plasma folates were less likely to experience GDM (OR 0.420 [95% CI 0.193–0.916]) (*p* = 0.029, Table 4).

**Table 4.** Association of maternal RBC folates and plasma folates with pregnancy complications risk.

	GH			GDM			PE		
Variables	OR	95% CI	*p* Vaule	OR	95% CI	*p* Vaule	OR	95% CI	*p* Vaule
RBC folates (nmol/L)									
906 − 1360		Reference			Reference			Reference	
<906	1.182	0.537 − 2.605	0.677	0.549	0.241 − 1.25	0.153	4.861	1.431 − 16.505	0.011
≥1360	0.645	0.321 − 1.297	0.219	0.755	0.373 − 1.526	0.433	1.193	0.376 − 3.781	0.764
Plasma folates (nmol/L)									
Q2		Reference			Reference			Reference	
Q1	1.275	0.595 − 2.734	0.532	1.125	0.526 − 2.4406	0.762	3.44	1.254 − 9.434	0.016
Q3	2.274	1.11 − 4.657	0.025	0.420	0.193 − 0.916	0.029	1.004	0.283 − 3.561	0.995

GH: Q3 (≥65.82), Q2 (38.81 − 65.82), Q1 (<38.81);

GDM: Q3 (≥60.09), Q2 (32.95 − 60.09), Q1 (<32.95);

PE: Q3 (≥63.99), Q2 (37.09 − 63.99), Q1 (<37.09).

## Discussion

In this study, we developed and validated a LC-MS/MS method for simultaneous quantitation of FA and 5-MTHF in plasma and RBCs, which displayed satisfactory analytical performance. Several LC-MS/MS methods for folates determination have been reported previously and were shown to be superior to the conventional methods (microbiological assays or folate-binding proteins assays) in terms of its efficiency and sensitivity, and was considered a better tool in evaluating the whole picture of folate metabolism [18,26,27].

The levels of FA and 5-MTHF in RBCs were both found increased with our patients as GWs went up, suggesting increased physiological demands to support maternal/fetal development. While in a Danish study, the RBC folate levels demonstrated a significant decline from 18 to 32 weeks of gestation [3]. This discrepancy may be attributed to a variety of factors, such as the timing of intervention, the amount of FA supplementation and population [28,29].

More importantly, our study showed a negative association between the plasma folates and GDM, suggesting a potential protective effect of folate supplementation in pregnancy. It is well known that FA supplements helps to reduce serum homocysteine (Hcy) concentrations [30]. However, the underlying mechanisms explaining the effect of folates on GDM development have not been elucidated. An epidemiological study conducted in Boston (USA) suggested that an imbalance of folate status or intake and vitamin B12 predisposed women to diabetes and their offspring to insulin resistance and adiposity and low birthweight [31].

It was previously found [32] that the women with lower concentrations of folates were more likely to develop PE. Similarly, in a meta-analysis including 14 studies showed that the supplementation of FA may improve placental implantation and finally played a key role in reducing the risk of PE [33]. Additionally, a Chinese study reported that FA supplementation and dietary folate intake during pregnancy are associated with a protective effect for PE and their risk reduction may vary by severity of PE [28]. In the two-stage model of the pathophysiology of PE, the supplementation of large doses of FA in early gestation was believed to work at both stages of PE development [34,35]. It has been demonstrated that FA played essential function in angiogenesis through a nitric oxide-dependent mechanism and the deficiency of FA may possibly induce apoptosis of human trophoblast cells, thus affecting trophoblast invasion and placental development [36–38]. Our findings and those previous studies may provide some insight into the possibility that testing for RBC folates rather than plasma folates during pregnancy was better for the prevention of PE.

There are several limitations associated with this study. For instance, a few important confounding factors, such as medication use or dietary supplementation of folates, physical activities, age and so on, were not included especially in the association analysis. In addition, the statistical power of our study was limited by the relatively small population size and was conducted in a single medical site in northern China. Considering the geographical and ethnic differences in folate concentrations [39], a multicenter-study with larger pregnant populations is warranted to confirm or explore the findings of this study.

## Conclusions

In conclusion, a laboratory-developed assay for simultaneous quantitation of the folates and metabolites in plasma and RBCs was validated with good performance. In addition, the concentrations of folates and metabolites were diversely associated with the pregnancy complications, such as GDM, GH and PE development. Our work undoubtedly provides insightful laboratory evidence for clinicians to make appropriate dietary and supplement recommendations throughout pregnancy to insure healthy birth outcomes.

## Supplementary Material

Supplemental Material

## Data Availability

The derived data supporting the findings of this study are included within the article and its supplemental materials. The raw data were generated at Beijing Obstetrics and Gynecology Hospital, Capital Medical University and are available upon reasonable request from the corresponding authors.
