# Supplementary material for: Diverse associations observed between pregnancy complications and RBC or plasma folates determined by an in-house developed LC-MS/MS method
Source: Ann Med. 2023 Oct 12;55(2):2265381. doi: 10.1080/07853890.2023.2265381 (PMC10572033; doi:10.1080/07853890.2023.2265381)
Supplement: Supplemental Material [file IANN_A_2265381_SM8708.zip › Supplementary Tables.docx]

| **Supplementary Table 1** Diagnosis criteria of pregnancy complications and adverse prenatal outcomes | |  |
| --- | --- | --- |
|  |  |  |
| Diseases | Definition |  |
|  |  |  |
| GH | Blood pressure elevation [systolic blood pressure≥140 mmHg or diastolic blood pressure+B12≥90 mmHg] at >20 weeks’ gestation in the absence of proteinuria (1). |  |
| GDM | In an oral glucose tolerance test (OGTT), every participant is requested for a 50 g glucose challenge test and serum glucose levels are assayed 1 h later. Subjects with positive results (glucose levels≥7.8 mmol/L) are required to undergo a 75 g OGTT. Serum glucose levels during OGTT are measured at 0, 1 and 2 h, respectively. The normal values are fasting glucose <5.1 mmol/L, 1-h glucose <10.0 mmol/L and 2-h glucose <8.5 mmol/L. GDM is diagnosed if one or more values equaled or exceeded the above thresholds (2). |  |
| PE | New-onset hypertension (systolic blood pressure≥140 mmHg or diastolic blood pressure≥90 mmHg) and new-onset proteinuria (300 mg of protein in 24 h or a urine protein/creatinine ratio of 0.3 mg/dl) after 20 weeks of gestation, in a previously normotensive woman (1). |  |
| GH: gestational hypertension; GDM: gestational diabetes mellitus; PE: preeclampsia. | |  |
| **References** 1.Hypertension in pregnancy. Report of the American College of Obstetricians and Gynecologists' task force on hypertension in pregnancy. Obstet Gynecol. 2013;122(5):1122–31. 2.World Health Organization. Diagnostic criteria and classification of hyperglycemia first detected in pregnancy: a World Health Organization Guideline. Diabetes Res Clin Pract. 2014;103(3):341–63. | |  |

| **Supplementary Table 2** Calibration and quality control concentrations | | | | |
| --- | --- | --- | --- | --- |
| Level | FA (nmol/l) | 5-MTHF (nmol/l) |  |  |
| A: whole blood |  |  |  |  |
| 1 | 0.57 | 1.09 |  |  |
| 2 | 1.13 | 2.18 |  |  |
| 3 | 2.27 | 4.35 |  |  |
| 4 | 11.33 | 21.78 |  |  |
| 5 | 28.25 | 54.50 |  |  |
| 6 | 56.75 | 108.75 |  |  |
| QC1 | 0.91 | 1.74 |  |  |
| QC2 | 4.53 | 8.71 |  |  |
| QC3 | 11.33 | 21.76 |  |  |
| B: plasma |  |  |  |  |
| 1 | 0.5 | 1 |  |  |
| 2 | 1 | 2 |  |  |
| 3 | 2.5 | 5 |  |  |
| 4 | 10 | 20 |  |  |
| 5 | 25 | 50 |  |  |
| 6 | 50 | 100 |  |  |
| QC1 | 2 | 4 |  |  |
| QC2 | 20 | 40 |  |  |
| QC3 | 40 | 80 |  |  |
| Concentration levels in different calibrators and quality control samples. FA, folic acid; 5-MTHF, 5-methyltetrahydrofolate; QC, quality control sample. | | | | |

| **Supplementary Table 3** The analytical and instrumental parameters of the LC-MS/MS method | | | | | | |
| --- | --- | --- | --- | --- | --- | --- |
| Analytes | Q1^a^, m/z | productions | Q3^b^, m/z | DP, V | CE, V |  |
| FA | 442.1 | quantifier | 295.3 | 90 | 30 |  |
|  | 442.1 | qualifier | 176.2 | 70 | 50 |  |
| ^13^C_5_-FA | 447.2 | IS | 295.3 | 80 | 20 |  |
| 5-MTHF | 460.3 | quantifier | 313.2 | 100 | 20 |  |
|  | 460.3 | qualifier | 194.2 | 70 | 40 |  |
| ^13^C_5_-5-MTHF | 465.3 | IS | 313.2 | 90 | 30 |  |

DP, declustering potential; CE, collision energy; IS, internal standard. Q1^a^, parent ion; Q3^b^, product ions including quantifier and qualifier.
