# Supplementary figures and images for: Diverse associations observed between pregnancy complications and RBC or plasma folates determined by an in-house developed LC-MS/MS method
Source: Ann Med. 2023 Oct 12;55(2):2265381. doi: 10.1080/07853890.2023.2265381 (PMC10572033; doi:10.1080/07853890.2023.2265381)

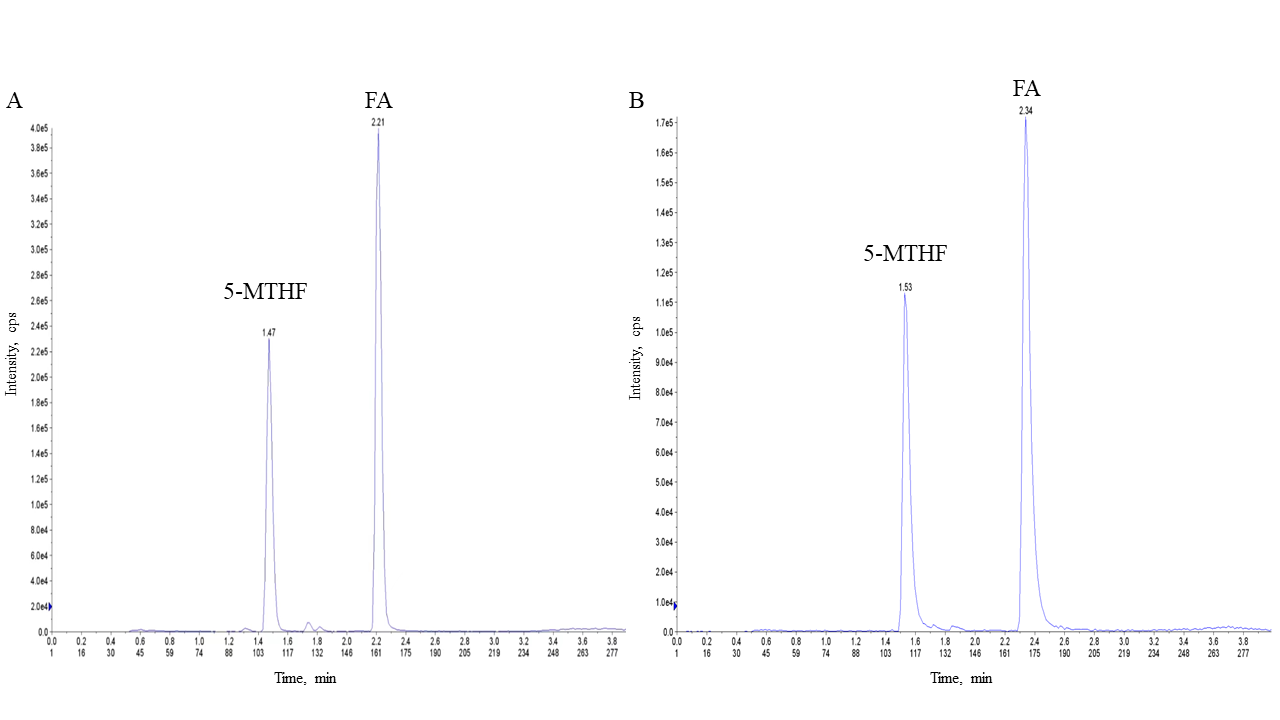

Supplement: Supplemental Material [file IANN_A_2265381_SM8708.zip › Supplementary Figure 1.tif]
